# Material and Process Tests of Heterogeneous Membranes Containing ZIF-8, SiO_2_ and POSS-Ph

**DOI:** 10.3390/ma15186455

**Published:** 2022-09-17

**Authors:** Daniel Polak, Maciej Szwast

**Affiliations:** Faculty of Chemical and Process Engineering, Warsaw University of Technology, Warynskiego 1, 00-645 Warsaw, Poland

**Keywords:** heterogeneous membranes, inorganic fillers, permeability, solubility, diffusivity

## Abstract

Heterogeneous membranes made of a polymer matrix and containing nano-metric fillers in their structure may present improved physicochemical and process properties compared to homogeneous membranes made only of polymer materials. Membranes made of a PEBAX^®^2533 block copolymer were tested with fillers such as ZIF-8, SiO_2_ and POSS-Ph being dosed to them. The material analysis and process tests indicate that these nanomaterials can be used as fillers for heterogeneous membranes. Chemometric analyses determined the influence of individual fillers on selected physicochemical properties of the materials which were used to produce the membranes. For specific concentrations of these fillers, improvement in the permeability and selectivity of the membranes, or at least in one of these parameters, was achieved. The greatest increase in permeability against the homogeneous membrane was obtained for membranes containing 10 wt% ZIF-8 (for CO_2_, an increase of 2.07 times; for CH_4_, 2.36 times; for N_2_, 3.08 times). In turn, the greatest increase in selectivity was obtained for the CO_2_/CH_4_ mixture for the membrane containing 5 wt% SiO_2_ (1.15 times), and for the CO_2_/N_2_ mixture for the membrane containing 2 wt% POSS-Ph (1.21 times).

## 1. Introduction

Gas separation is a significant industrial process. The need for the use of pure gases is recognized virtually in every industry. These gases are used as energy sources, reagents or inert gases. However, the individual gases are not found in nature as pure components, so they need to be separated or isolated from mixtures. For example, methane is obtained from a mixture of CH_4_/N_2_ or CH_4_/CO_2_, nitrogen or oxygen is taken from the atmospheric air, helium is isolated from natural gas, etc. [1,2,3,4].

Gas mixture separation processes are carried out by a number of methods, including absorption [5,6], adsorption [7,8], cryogenic techniques [9,10], molecular screening [11,12] and membrane techniques [13,14,15]. Naturally, each of these methods has its advantages and disadvantages. However, with the development of polymer technologies, membrane techniques, in particular those using hollow-fiber membranes, appear to become increasingly important in industrial mixture separation processes [16].

The advantages of using membranes to separate components of gas mixtures include relatively low system preparation and operational costs, high purity of product streams obtained, the possibility of scaling up and the absence of waste streams, in particular those that are harmful to health and the environment [17,18].

Membranes designed for gas separation can be made of various materials, but polymer membranes are the most common [19]. This is mainly due to the availability of these materials, their relatively low cost and the ease of processing and scaling up the process that polymer membranes are engaged in. With the use of polymer membranes, high-purity products can be obtained with satisfactory streams of these products. However, for polymer membranes, a certain relationship has been identified that is currently considered to be a limitation in the applicability of this type of membrane. It appears that it is hard, but possible, to achieve both a high separation coefficient (product purity) and a high-value product stream (process efficiency) at the same time [20,21,22]. It is desirable to achieve high values for both of these process parameters at the same time to make the process cost-effective. Sometimes, for some gas mixtures, the existing limitation prevents the use of polymer membranes for industrial gas separation processes. The best example is a mixture of CH_4_ and N_2_, where the separation factor is about 1. This prevents the use of polymeric membranes in an important industrial process: the denitrification of natural gas. Studies have been conducted extensively, aiming to overcome these limitations while using low-cost polymer materials to create a membrane. To overcome the natural limitations posed by polymer materials, micro- or nanofillers from other materials have been introduced into the structure of polymer membranes [23,24].

Membranes made of polymer materials and containing fillers from other materials are called heterogeneous or mixed-matrix membranes (MMMs) [24,25]. The fillers used in heterogeneous membranes may include carbon nanotubes (CNTs) [26,27], ZIF and POSS structures [28,29,30,31], as well as oxides of various elements (such as SiO_2_, TiO_2_, Al_2_O_3_ or graphene oxide) [32,33]. The fillers can be incorporated into the polymer structure by the bulk modification method [34,35], such as in this paper, as well as by other methods, such as decoration [36,37].

The use of fillers in heterogeneous membranes replaces the mechanism of solubility and diffusion transport [38] with another, depending on the porosity or nonporosity of the filler applied [39,40]. Regardless of the type of filler used, it is important for these membranes to be made properly to ensure that such membranes work correctly. The process parameters, i.e., permeability and separation coefficient, of these membranes are affected by the degree and uniformity of dispersion of the fillers in the polymer matrix and the interaction between the fillers and the polymer chains [41]. The choice of a polymer as a matrix in a heterogeneous membrane and fillers appears to be crucial in achieving the expected properties of the membrane to be produced.

One interesting polymer that can be used to create a membrane for gas separation is polyether block amide. This polymer has interesting separation properties, particularly in relation to CO_2_, which, despite its large molecule size, is preferentially transported through a membrane. To improve its properties, the matrix of this polymer can be enriched with fillers. We chose porous (ZIF-8 and POSS-Ph) and nonporous (SiO_2_) fillers for testing. In the case of porous fillers, the expected effect is the molecular sieving effect—smaller gas molecules will pass through the filler structure, while larger molecules will have to extend their diffusion path, which in turn will affect the separation coefficient of the new membrane for a given gas pair. On the other hand, for nonporous fillers, the expected effect is related not only to the extending of the diffusion path for all gas components, but also to structural changes within the polymer chains and the resulting new free volumes in the polymer. This is the objective pursued in the tests described herein. This article presents the results of the tests of heterogeneous membranes made of polyether block amide as well as ZIF-8, SiO_2_ and POSS fillers, respectively. Both the results of structural and physicochemical tests (SEM, EDX, FTIR, DSC and TGA) and the results of measurements of the membrane process parameters (permeability, diffusion coefficient, solubility) are presented. We chose three gases for process testing: CO_2_, N_2_ and CH_4_. Pairs of such gases are common in the environment and industry. CO_2_ and CH_4_ are the main components of biogas, while CO_2_ and N_2_ are the main components of flue gases. The enrichment of biogas and the purification of greenhouse gases from flue gases is a serious challenge for industrial processes, including membrane processes. The CO_2_ obtained in these separation processes can, for example, be used to produce methane in a process using the power-to-gas concept.

The aim of this paper was to collect information on the influence of various fillers on the properties of heterogeneous membranes (mixed-matrix membranes). While there are reports in the literature on gas separation membranes made of Pebax^®^2533 and ZIF-8 [42,43], it is difficult to find information about gas separation membranes made of this copolymer and SiO_2_ or POSS-Ph fillers. Moreover, in this paper, an attempt was made to link the results of chemometric studies with the process properties of membranes.

## 2. Materials and Methods

### 2.1. Polymer and Fillers

**Figure 1:**
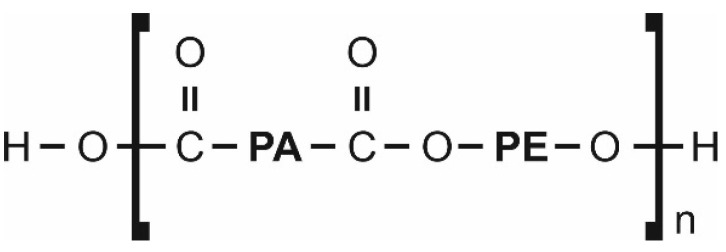
Chemical structure of polyether block amide (Pebax^®^2533); PA—polyamide group, PE—polyether group.

Polyether block amide under the trade name Pebax^®^2533 (Arkema, France) was used to produce heterogeneous membranes (Figure 1). This copolymer is often used in membrane gas separation tests [44,45,46]. As it has been mentioned, this polymer has interesting separation properties, particularly in relation to CO_2_, which, despite its large molecular diameter in comparison to such gases as O_2_, N_2_, CH_4_, H_2_ and He [47], is preferentially transported through a membrane. Furthermore, this copolymer is relatively inexpensive and easily processed. It dissolves at low temperatures (<100 °C) in solvents such as ethanol, propanol, butanol and N-Methyl-2-pyrrolidone (NMP). In these tests, 2-Butanol (Sigma-Aldrich, Poznan, Poland) is used as a solvent.

ZIF-8 (Basolite Z1200 by BASF, Sigma-Aldrich, Poland), SiO_2_ (nanopowder 10–20 nm, Sigma-Aldrich, Poland) and POSS-Ph (PSS-Octaphenyl substituted, Sigma-Aldrich, Poland) particles were used to fill the polymer matrix.

**Figure 2:**
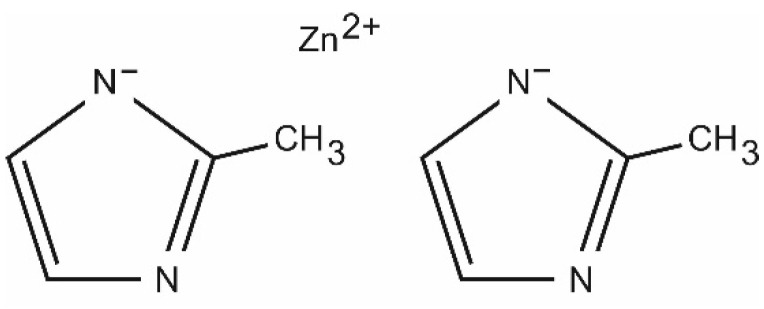
Chemical structure of ZIF-8.

ZIF-8 particles, Figure 2, are porous organometallic structures in which metallic ions, e.g., Co^2+^, Cu^2+^ or Zn^2+^, are combined with N atoms contained in imidazolinones. They form a tetrahedral structure [48].

SiO_2_ particles, with commonly known chemical structure, are nonporous nanoparticles with hydrophilic properties [49].

**Figure 3:**
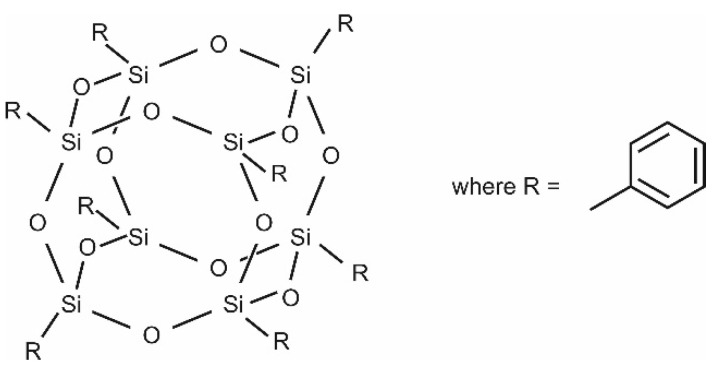
Chemical structure of POSS-Ph.

POSS-Ph (Phenyl POSS) particles, Figure 3, are polyhedral oligomeric silsesquioxanes that form spatial porous structures in which various substituents may be attached to SiO_1.5_ groups [50]. 

### 2.2. Membrane Preparation

The membranes were produced by a dry inversion phase separation method (DIPS) [51]. The membranogenic solution was prepared as follows: A weighed portion of Pebax^®^2533 copolymer granulate was added to the measured amount of solvent (2-Butanol) and heated to 80 °C to obtain a solution of 7 wt% polymer in the solvent. Then, stirring vigorously, this solution was left at this temperature for at least 24 h, until the polymer completely dissolved and a homogeneous solution was obtained. After this time, maintaining the set temperature and stirring continuously in a vigorous way, a weighed amount of either ZIF-8, SiO_2_ or POSS-Ph filler was added gradually. By adding different weights of fillers, membranogenic mixtures with different filler contents (referring the weight of fillers to the weight of the polymer) and, consequently, membranes with different filler contents in the polymer matrix were obtained. The mixture was left at the set temperature for another 24 h, still stirring, but less vigorously. After this time, the mixture was transferred to an ultrasonic bath for several minutes. The next step was to pour out and evenly distribute the membranogenic mixture on a heated glass pane using a casting knife. The prepared membrane was left covered and on the heated glass pane until the solvent evaporated and the membrane solidified (24–48 h). The prepared membrane was then removed from the glass into an ultrapure water bath. After drying, the membrane was ready for testing.

### 2.3. Analytical Techniques

In order to describe the membranes produced and to examine their physicochemical and process properties, they were subjected to a series of tests using various measurement techniques.

Scanning electron microscopy (SEM) was used to assess the quality of the membranes produced and, in particular, the presence of agglomerates or defects in the membrane structure was observed. In addition, this technique was used to measure the thickness of the membrane, which was determined as the average of 9 thickness measurements made in different places of the sample. The PhenomPro (PhenomWorld, Eindhoven, The Netherlands) device was used for the SEM analysis, and the charge reduction sample holder was applied to test non-conductive samples without the need for sputtering.

The EDX (energy-dispersive X-ray spectroscopy) technique was used to confirm the presence of new elements, due to the fillers being added, that were not present in the polymer, on the membrane surface. The JSM-IT500LA device with the dry SD30 EDX detector (JEOL, Freising, Germany) was used for the EDX analysis.

Fourier transform infrared spectroscopy (FTIR) with the attenuated total reflectance (ATR) module was used to identify the function groups on the membrane surface that may occur as a result of adding different fillers to the polymer matrix. The tests were conducted with the Nicolet iS10 (Thermo Scientific, Waltham, MA, USA) spectrometer. The sample was scanned 32 times within the wave numbers 4000–800 cm^−1^.

Differential scanning calorimetry (DSC) was used to investigate changes in the thermal effects connected with the polymer’s phase changes. The glass point and melting point were determined to assess the effect of inorganic fills on the heterogeneous membrane structure. The presence of an inorganic phase can cause an increase in temperatures, which will be linked to rigidity of the polymer chains. In turn, a decrease in their values may result from breaking the bonds between the polymer chains, which may lead to an increase in their mobility or greater free-space volume. In addition, on the basis of the determined values of the melting enthalpy, it is possible to determine the degree of crystallinity for the PE (polyether) and PA (polyamide) groups as well as the total degree of crystallinity. The DSC analysis was performed using the TA Instruments Q2000 (TA Instruments, New Castle, DE, USA). The samples were analyzed in an argon atmosphere within the temperature range −90 °C to 200 °C at a heating rate of 10 °C/min. The weight of the sample was 10 mg.

Thermogravimetric analysis (TGA) was used to investigate the presence of the solvent and water in the structure of the membrane once it was produced. This method also examines the influence of an inorganic filler on the thermal stability of the sample. We found that a sample begins to lose thermal stability when it loses 5 wt% of its initial weight, and a complete loss of stability is recorded with a loss of 10 wt% of the initial sample weight. Therefore, the temperatures at which the weight loss was 5 wt% and 10 wt% were measured. TGA analysis was performed using TGA/DSC 3+ (Mettler Toledo, Columbus, OH, USA). The samples were analyzed in an argon atmosphere within the temperature range 25 °C to 600 °C at a heating rate of 10 °C/min. The weight of the sample was 10 mg.

In order to examine the process properties of the membranes produced, i.e., their permeability (*P*), diffusion coefficient (*D*), solubility (*S*) and ideal selectivity (*α*), the time-lag method [52] was used, a modified version of which we presented in our previous works [44]. A membrane with a diameter of 80 mm (i.e., area of approx. 50 cm^2^) was placed in a special holder and tested using selected pure gases. The tests were carried out at a pressure of 4 bar and at a temperature of 40 °C.

## 3. Results and Discussions

The tests were conducted for a wide range of filler concentration values in the polymer matrix. The results for the selected concentrations are shown below. The upper limit of the concentration values (10 wt% for ZIF-8 and SiO_2_ and 8 wt% for POSS-Ph) is the concentration above which the membranes produced were lacking mechanical strength and had non-recurring physicochemical parameters. The first of the concentration values presented (2 wt% for ZIF-8 and SiO_2_ and 0.75 wt% for POSS-Ph) was selected as the one for which there was a noticeable increase in permeability and selectivity compared to the homogeneous membrane.

The following section shows the results obtained for the measurement techniques and methods described above.

### 3.1. Scanning Electron Microscopy (SEM) and Energy-Dispersive X-ray Spectroscopy (EDX)

**Figure 4:**
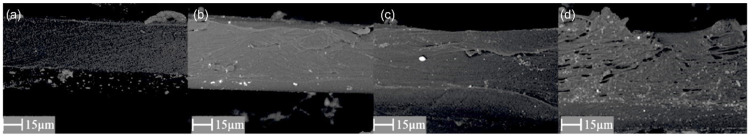
SEM image of membrane cross section with ZIF-8 added; (**a**) PEBAX 2533 + 0 wt% ZIF-8, (**b**) PEBAX 2533 + 2 wt% ZIF-8, (**c**) PEBAX 2533 + 5 wt% ZIF-8, (**d**) PEBAX 2533 + 10 wt% ZIF-8.

**Figure 5:**
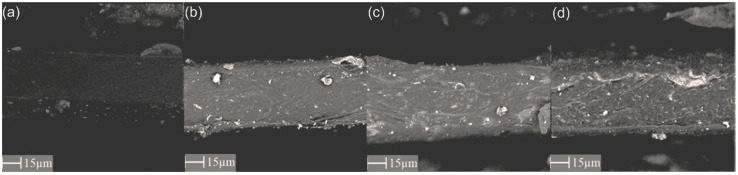
SEM image of membrane cross section with SiO_2_ added; (**a**) PEBAX 2533 + 0 wt% SiO_2_, (**b**) PEBAX 2533 + 2 wt% SiO_2_, (**c**) PEBAX 2533 + 5 wt% SiO_2_, (**d**) PEBAX 2533 + 10 wt% SiO_2_.

**Figure 6:**
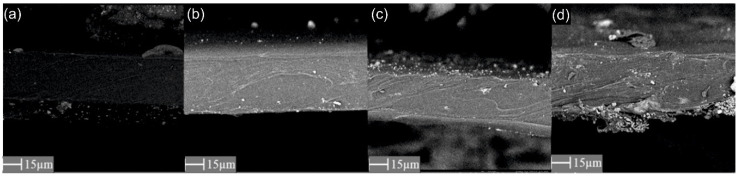
SEM image of membrane cross section with POSS-Ph added; (**a**) PEBAX 2533 + 0 wt% POSS-Ph, (**b**) PEBAX 2533 + 0.75 wt% POSS-Ph, (**c**) PEBAX 2533 + 2 wt% POSS-Ph, (**d**) PEBAX 2533 + 8 wt% POSS-Ph.

**Figure 7:**
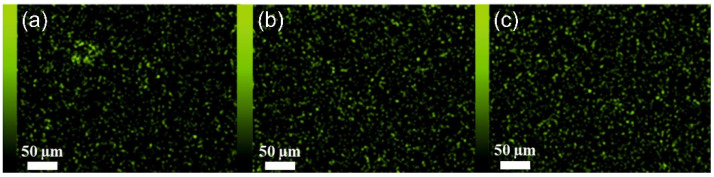
Surface of membranes containing ZIF-8. Mapped Zn; (**a**) PEBAX 2533 + 2 wt% ZIF-8, (**b**) PEBAX 2533 + 5 wt% ZIF-8, (**c**) PEBAX 2533 + 10 wt% ZIF-8.

**Figure 8:**
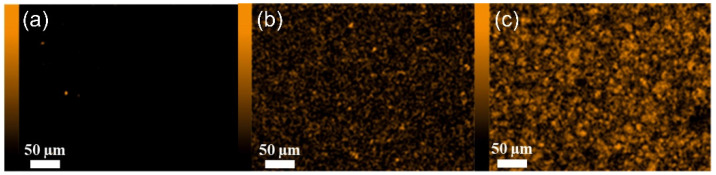
Surface of membranes containing SiO_2_. Mapped Si; (**a**) PEBAX 2533 + 2 wt% SiO_2_, (**b**) PEBAX 2533 + 5 wt% SiO_2_, (**c**) PEBAX 2533 + 10 wt% SiO_2_.

**Figure 9:**
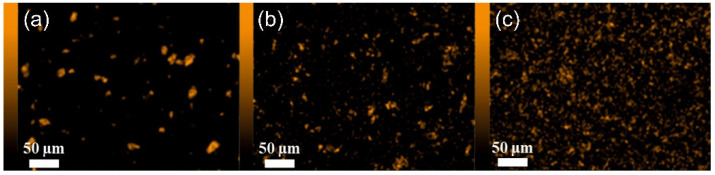
Surface of membranes containing POSS-Ph. Mapped Si; (**a**) PEBAX 2533 + 0.75 wt% POSS-Ph, (**b**) PEBAX 2533 + 2 wt% POSS-Ph, (**c**) PEBAX 2533 + 8 wt% POSS-Ph.

Analysis of the SEM images (Figure 4, Figure 5 and Figure 6) confirms the presence of the filler particles in the membrane structure, ZIF-8, SiO_2_ and POSS-Ph, respectively. However, these images do not prove that the dispersion of the particles in the entire membrane is homogeneous. However, we can see few agglomerates of those particles which are not desirable at the stage of membrane production but are inevitable. On the other hand, the conclusion of the EDX analysis (Figure 7, Figure 8 and Figure 9) is that the filler particles are quite homogeneously dispersed in the polymer structure. This is demonstrated by the elements, as indicated by the detector, that are only contained in the filler particles, and are not present in the polymer. The two photos (Figure 5a and Figure 6a) are surprising. They show EDX results for membranes with the lowest SiO_2_ and POSS-Ph contents, respectively. The first one shows practically no fillers on the surface of the sample, while the second one shows a low homogeneity of dispersion. SEM pictures of membrane cross sections, in turn, do not seem to confirm this observation. The observed effects in mentioned figures are probably due to the fact that, with a small amount of filler in the polymer matrix, these fillers have hidden under the membrane surface and are mostly covered by the polymer layer. This effect may also be related to the sensitivity of the EDX detector towards Si atoms at a low concentration [53].

### 3.2. Fourier Transform Infrared Spectroscopy (FTIR) 

**Figure 10:**
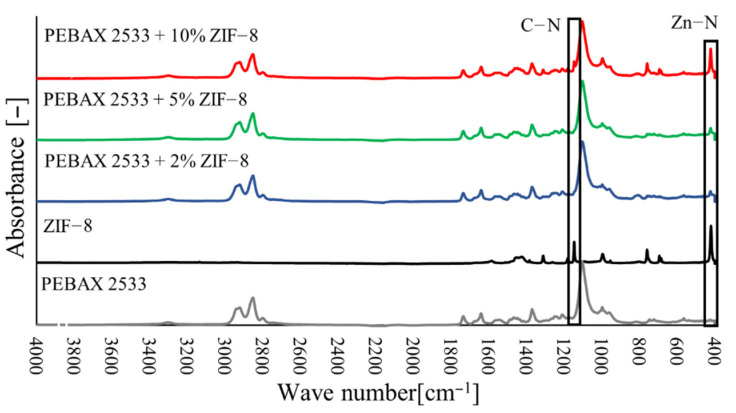
FTIR analysis of membranes containing ZIF-8 in different concentrations.

**Figure 11:**
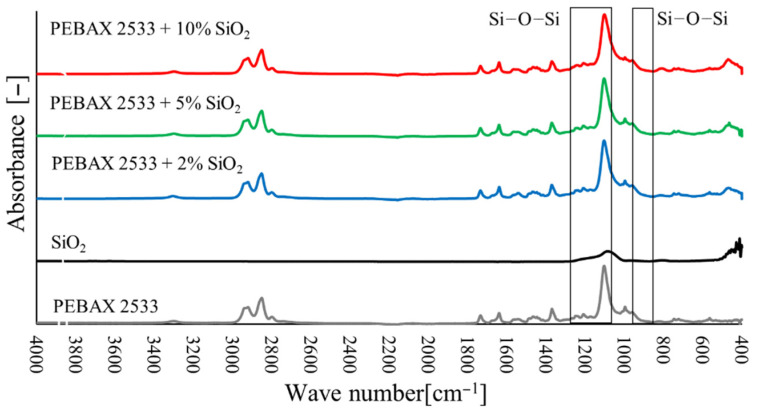
FTIR analysis of membranes containing SiO_2_ in different concentrations.

**Figure 12:**
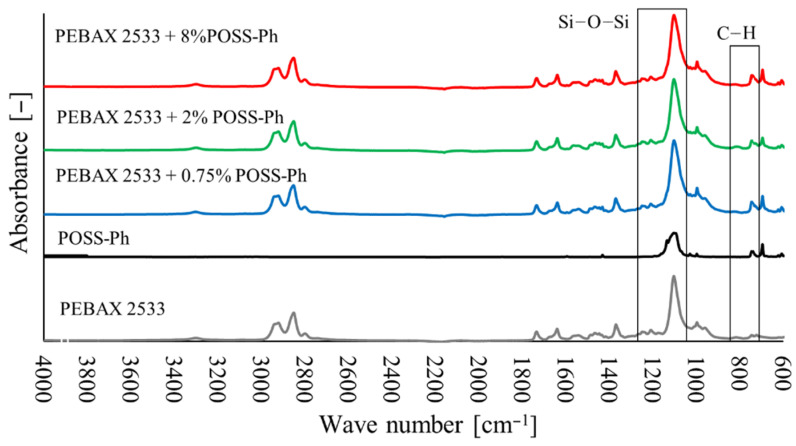
FTIR analysis of membranes containing POSS-Ph in different concentrations.

The FTIR tests (Figure 10, Figure 11 and Figure 12) also confirmed the appearance of new bonds in the material structure that are not present in the polymer material. Thus, for membranes containing ZIF-8, the typical absorption bands correspond to the vibrations of the Zn–N (421 cm^−1^) bond and the vibrations of bonds that occur in imidazole ligand in the range of wave numbers 500–1500 cm^−1^ (Figure 10). The strong peak that occurs with wave number 1146 cm^−1^ corresponds to the vibrations of the C–N bond. For membranes with 2 wt% and 5 wt% ZIF-8 content, the peak corresponding to the C–N bond is invisible, due to too low intensity of absorbance (too low concentration of ZIF-8 particles on the material surface) or due to overlapping with the spectrum that is typical of the PEBAX^®^2533 material. However, a peak corresponding to the Zn–N bond is clearly visible, with its intensity growing as the filler concentration increases. On the other hand, for SiO**2** membranes, peaks that are typical of SiO_2_ were observed which correspond to asymmetric Si–O–Si (1083 cm^−1^) bonds and symmetric Si–O–Si (800 cm^−1^) bonds (Figure 11). Unfortunately, these peaks overlap with the peaks that are typical of the groups present in the Pebax^®^2533 copolymer structure, and only a slight widening of the peak is exposed in heterogeneous membrane tests. However, in the case of membranes containing POSS-Ph, new peaks may be observed for membranes containing POSS-Ph in the wavelength range 730–860 cm^−1^ (Figure 12). These peaks correspond to the C–H_aromat._ or C–H di- or monosubstituted bonds which occur in the aromatic structures. In the POSS-Ph structure, a peak that is typical of wave number 1091 cm^−1^ can also be observed, corresponding to asymmetric Si–O–Si bonds. However, this peak overlaps with peaks that are typical of groups occurring in the Pebax^®^2533 copolymer particle structure, and it is difficult to confirm the presence of new groups on the membrane surface on its basis.

### 3.3. Differential Scanning Calorimetry (DSC), Thermogravimetric Analysis (TGA) and Process Parameters (P, D, S, α)

**Figure 13:**
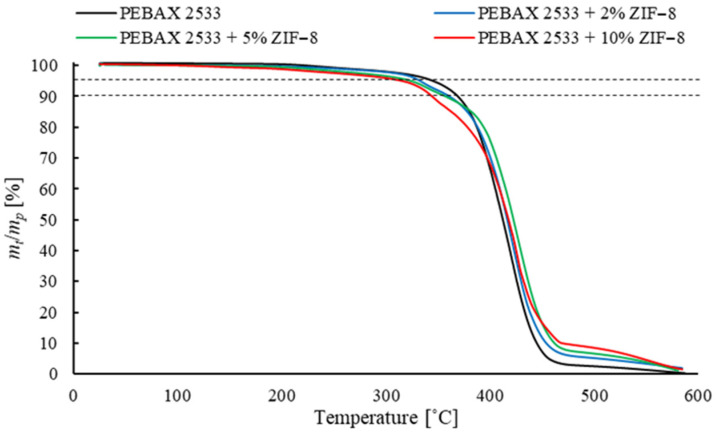
TGA analysis of membranes containing ZIF-8 in different concentrations.

**Figure 14:**
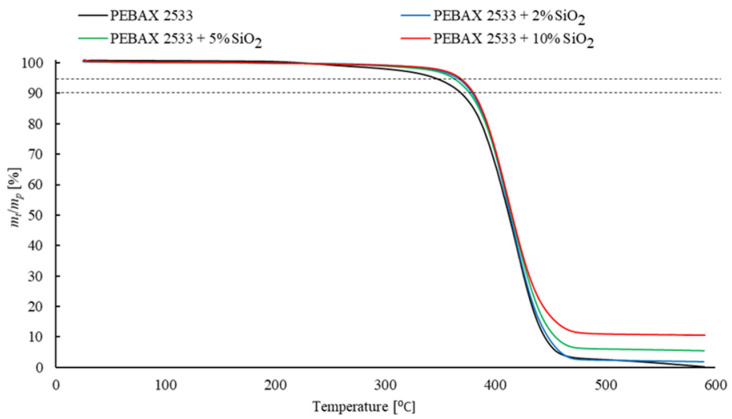
TGA analysis of membranes containing SiO_2_ in different concentrations.

**Figure 15:**
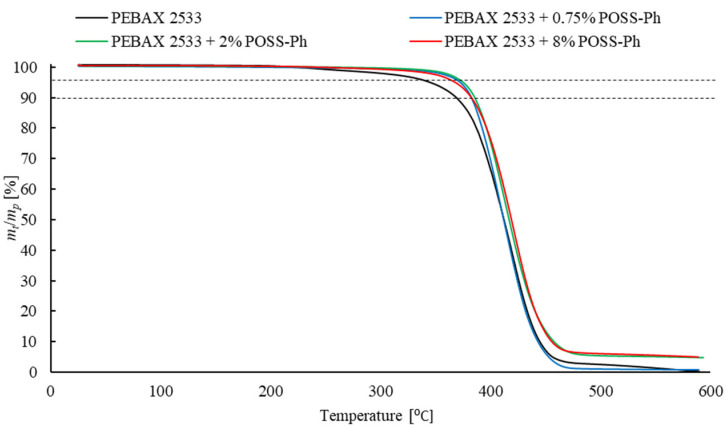
TGA analysis of membranes containing POSS-Ph in different concentrations.

**Figure A1:**
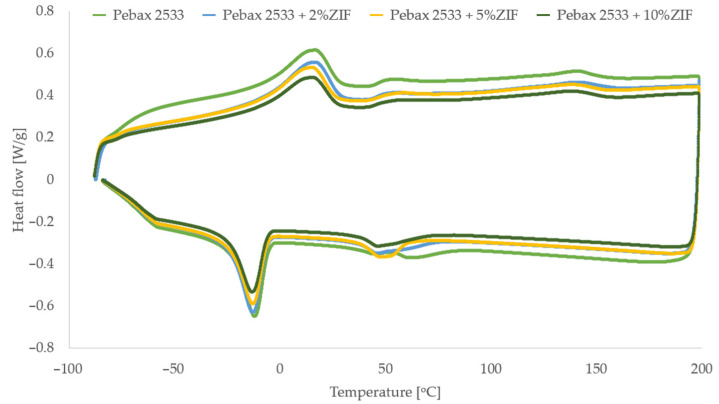
DSC thermogram for membranes containing ZIF-8 in different concentrations.

**Figure A2:**
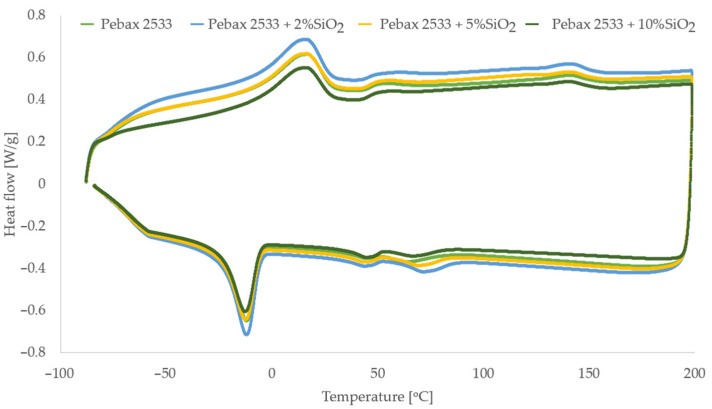
DSC thermogram for membranes containing SiO_2_ in different concentrations.

**Figure A3:**
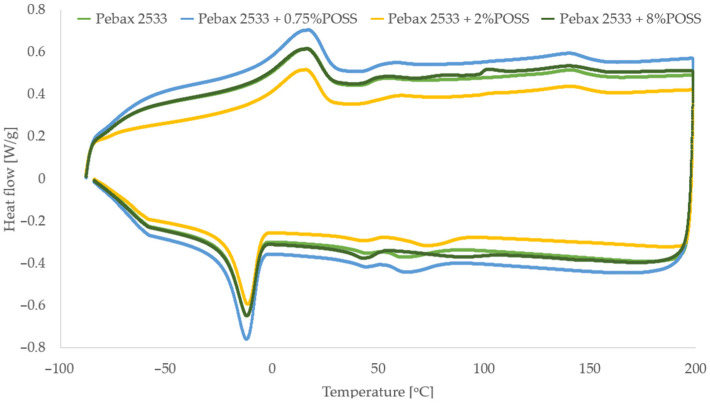
DSC thermogram for membranes containing POSS-Ph in different concentrations.

The results of the DSC and TGA tests cannot be interpreted without a direct reference to the test results of the process parameters of the analyzed membranes. Some of results are presented below (Figure 13, Figure 14 and Figure 15 and Table 1, Table 2, Table 3, Table 4, Table 5, Table 6, Table 7, Table 8 and Table 9) and some of additional results are presented in Appendix A (Figure A1, Figure A2 and Figure A3 and Table A1, Table A2, Table A3, Table A4, Table A5, Table A6, Table A7, Table A8, Table A9, Table A10, Table A11 and Table A12). 

In Table 1, Table 2 and Table 3, the following symbols are used: 

  *T_m_*—melting temperature;

  *H_m_*—melting enthalpy;

  *X_c_*—crystallinity;

  PE—polyether group (amorphous phase);

  PA—polyamide group (crystalline phase).

The thermograms can be found in Appendix A Figure A1, Figure A2 and Figure A3.

In presenting the results of the gas permeation measurements of the developed membranes, a barrer unit was used, which is a non-SI unit, but is generally accepted in membrane-related literature. The conversion into SI units can be done as follows:

(1) $$1\;\mathrm{barrer}=3.35\;\cdot 10^{-16}\;\frac{\mathrm{mol}\;\cdot \;m}{m^{2}\;\cdot \;s\;\cdot \;\mathrm{Pa}}$$

### 3.4. Membranes with ZIF-8

The results for membranes containing ZIF-8 are shown in Table 1, Table 4, Table 5, Table A1, Table A4, Table A5 and Table A6 and Figure 13 and Figure A1. Values of membrane permeability coefficients for the test gases *P*_CO2_, *P*_CH4_ and *P*_N2_ and ideal selectivity coefficients of CO_2_ in relation to CH_4_ *α*_CO2/CH4_ and N_2_ *α*_CO2/N2_ for the test membranes were determined. On the basis of the results, it can be concluded that the permeation coefficient for all the gases grows with the increase in concentration of ZIF-8. In general, this has also been observed by other authors [42]. However, it should be noted that above the specified concentration, there is a decrease in the value of the ideal selectivity coefficient of CO_2_ in relation to other gases, particularly in relation to N_2_. This effect may indicate the formation of agglomerates and free spaces around the filler particles. The increase in both the permeation coefficient of CO_2_ and ideal selectivity occurs only for the membranes with low ZIF-8 concentrations. This may suggest that there is a low intensity of adverse effects in the membrane structure only for low filler concentrations. The exact explanation of the changes in the process properties of membranes containing ZIF-8 requires an analysis of their influence on the solubility and diffusion of the gases. On the basis of the results, it can be concluded that solubility of the gases improves as the concentration of ZIF-8 increases. It should be noted that the values of *α^S^*_i/j_ coefficients also increase. This effect suggests that the addition of a filler improves the solubility of CO_2_ more intensively than N_2_ or CH_4_. This is connected with the imidazole ligands that are present in the ZIF-8 structure. Between these groups and the CO_2_ molecules, proton–donor interactions occur, which positively affect the sorption of CO_2_ on the membrane surface. On the other hand, the increase in the solubility of other test gases—as well as, to some extent, CO_2_—results from the expansion of the membrane surface, which increases the gas–membrane contact surface. The expansion of the outer layer of the material is related to the presence of filler particles and their agglomerates at the membrane surface. Some authors also note an increase in the specific surface area of heterogeneous membranes and measure it by the surface roughness [54]. In addition, micro-cracks are formed around the filler particles, which is due to the difference in thermal expansion of the polymer and inorganic additive particles, resulting in the formation of micro-cracks when the solvent is evaporated. As the ZIF-8 concentration grows, increased *D*_i_ and reduced *α^D^*_i/j_ can be observed. The first effect is probably due to the formation of free spaces around the filler particles and their agglomerates, which reduces the resistance to gas penetration. The second effect, i.e., the reduced coefficient *α^D^*_i/j_, is due to the fact that the emerging zones are larger in size than the kinetic diameters of the test gas molecules, which results in equalizing the transport rate of the various gases through the membrane. In addition, the increase in the distance between the polymer chains due to the presence of filler particles reduces the effect of the phenomenon responsible for the privileged transport of CO_2_ by materials made of Pebax^®^2533. It should also be noted that for membranes containing ZIF-8 with 10 wt% concentration, coefficients *α^D^*_i/j_ are drastically reduced. This is due to the intense formation of agglomerates and the delamination of the membrane structure, as can be seen in the SEM images. The adverse effects resulting from the presence of ZIF-8 particles are also confirmed by the TGA results. Based on the drop in temperatures *T_d_*_5%_ and *T_d_*_10%_ that are typical of modified membranes—as compared to temperatures of membranes made of copolymer only—a deterioration in the thermal strength of materials containing ZIF-8 can be observed. The decrease in these temperatures is connected with the abovementioned adverse effects of agglomerates and free spaces being formed around the filler particles, which in turn results from poor interactions between the filler particle and the polymer. These effects reduce the thermal resistance of the material. The formation of free spaces or breaking of bonds between the polymer chains as a result of the presence of ZIF-8 particles is also confirmed by the results of the DSC analysis. It can be noted that the addition of ZIF-8 reduces both the melting point and the crystallinity degree of the PE (*T_m_*_PE,_ *X_C_*_PE_) and PA (*T_m_*_PA,_ *X_C_*_PA_) groups and, thus, also the total crystallinity of the *Xc* material. The intensity of these changes grows as the ZIF-8 concentration increases. Lower temperature values and reduction in the crystallinity degree of the material results from the breaking of the hydrogen bonds between the polymer chains or hydrogen bonds interacting with filler. Similar observations can be found elsewhere [55]. In addition, reduced values of these parameters may indicate that physical interactions between the polymer chains and filler particles do not significantly affect the structural properties of the material and, thus, the transport of gases. In conclusion, the increased diffusion of the test gases through membranes containing ZIF-8 is the result of these adverse phenomena. Theoretically, specific gas–filler or polymer–filler interactions, in this case, do not occur or their effect on the diffusion of gases is negligible. On the other hand, the decrease in membrane selectivity, which takes into account only differences in gas diffusion, is due to the formation of free spaces around filler particles and within their agglomerates. These zones occupy a non-selective space. Similar effects were noticed and similar conclusions, but for a different polymer and filler, were made in [56].

### 3.5. Membranes with SiO_2_

The relevant results for the membranes containing SiO_2_ in their structure are shown in Table 2, Table 6, Table 7, Table A2, Table A7, Table A8 and Table A9 and Figure 14 and Figure A2. On the basis of the results, an increase in the permeation coefficients of the test gases and in the ideal selectivity to a certain value of the filler concentration can be identified. A decrease in both the permeability and selectivity of the membrane can then be observed. These effects indicate that the adverse phenomena due to the presence of a diffusion phase have a significant effect only above a specified concentration of SiO_2_. For a lower content of the additive, the defects may not be present or their adverse effect on the process properties of the membrane is set off by improved CO_2_ transport in relation to other test gases. The exact explanation of the changes in the process properties of the membranes requires the determination of the influence of SiO_2_ on the solubility and diffusion of the gases. The values of the solubility coefficients of the test gases *S*_i_ and the selectivity coefficients related to the differences in the solubility of gases *α^S^*_i/j_ were analyzed. It is concluded that as the concentration of SiO_2_ increases, gas solubility improves. It should be noted that the values of *α^S^*_i/j_ coefficients also increase. This effect suggests that the addition of a filler improves the solubility of CO_2_ more intensively than N_2_ or CH_4_. This is due to the presence of -OH groups, which are responsible for bonding the Pebax^®^2533 polymer with partially hydrated SiO_2_ [57]. Interactions occur between CO_2_ molecules and -OH groups that improve CO_2_ sorption. Furthermore, in the case of this filler, similar to membranes containing ZIF-8, the specific surface of the membranes is increased. On the other hand, based on the results of diffusion coefficients and coefficients *α^D^*_i/j_, a decrease in diffusion rates of the test gases and statistically insignificant changes in the coefficient *α^D^*_i/j_ for membranes with SiO_2_ content of 2 wt% and 5 wt% are identified. The decrease in diffusion coefficients and the lack of changes in coefficients *α^D^_i/j_* indicate that there is a rigidity effect of the chains around the filler particles. This defect is a result of the interaction between the above -OH and -NH groups in the polyamide part of the copolymer and the polar PEO group. Such interactions have been reported with a different type of membrane [58]. Interactions with -NH groups are confirmed by the results of the DSC analysis, on the basis of which an increase in the crystallinity degree of the PA *X_C_*_PA_ areas can be observed. However, for PE groups, there is a drop in the *X_C_*_PE_ crystallinity degree value and the melting point *T_m_*_PE_, as well as in the total crystallinity of the *Xc* material. These changes should cause an increase in the diffusion of gases, but this is not confirmed by the obtained values of *D*_i_. Thus, the decrease in the rate of diffusion of gases can be connected with the extended distance that the gas molecule has to cover, due to the presence of a nonporous filler. This effect also explains the fact that the coefficient *α^D^*_i/j_ did not change for a membrane with 2 wt% and 5 wt% SiO_2_ content. However, it should be noted that for materials containing 10 wt% nano-SiO_2_, the crystallinity degree of the PA group decreases in relation to other structures. This may indicate that the hydrogen bonds break between the polymer chains. This defect may explain the decrease in coefficients *α^D^*_i/j_ for higher concentrations of this filler. The free zones forming in the structure of the filler agglomerates are larger in size than the kinetic diameters of the test gas molecules, which results in equalizing the transport rate of the various gases through the membrane, as observed in [59]. It should also be noted that for membranes with 10 wt% SiO_2_ content, a decrease occurs both for the values of coefficients *α^D^_i/j_* as well as for coefficients of CO_2_ diffusion and permeation. This may be due to the interactions between the filler particles and the molecules of this gas, which prevent its diffusion through the membrane. On the other hand, the TGA analysis shows that the presence of heat-resistant SiO_2_ particles also improves the thermal strength of the material, which is associated with increased temperatures of the sample decomposition. The lack of decrease in the thermal strength of the material even for a large filler content may indicate a relatively low influence of SiO_2_ on the mechanical properties of the membrane. In conclusion, the presence of SiO_2_ particles in the polymer matrix reduces the crystallinity degree of the membrane, which should increase the diffusion of the gases. However, it also causes the diffusion distance to extend, which turns out to be a factor that limits the rate of gas transport, while the decrease in the diffusion coefficient for CO_2_ and the diffusion coefficient for CH_4_ and N_2_, respectively, may result from the emergence of agglomerates and from the interactions between the molecules of this gas and filler particles. Similar effects were noted for different polymer by other authors and are presented in [60]. These effects are only visible for high filler concentrations.

### 3.6. Membranes with POSS-Ph

The relevant results for membranes containing POSS-Ph in their structure are shown in in Table 3, Table 8, Table 9, Table A3, Table A10, Table A11 and Table A12 and Figure 15 and Figure A3. The analysis of the permeability coefficients of the test gases and the selectivity coefficients of CO_2_ in relation to CH_4_ and N_2_ for the test membranes shows an increase in CO_2_ permeability through membranes with 0.75 wt% and 2 wt% filler content. For CH_4_ and N_2_, this change is slight compared to CO_2_, resulting in a noticeable improvement in membrane selectivity. For higher concentrations of the additive, the permeability of the test gases decreases and the separation properties of the materials deteriorate. For similar polymer and other POSS fillers, analogous effects were observed [30]. This type of adverse change in the properties of the membrane may be associated with the rigidity of the chains around the filler particles and the extension of the diffusion distance. In addition, a clear reduction in membrane selectivity of 8 wt% POSS-Ph content may indicate that agglomerates are formed, while the improved separation properties of the membrane in lower concentrations of the additive are probably due to the increased CO_2_ solubility. The exact explanation of the phenomena involved requires the determination of the changes in the solubility and diffusion of the gases through the membrane, depending on the amount of filler that is used to produce it. The resulting values of the solubility coefficients of the test gases *S*_i_ and the selectivity coefficients related to the differences in the solubility of the gases *α^S^*_i/j_ make it possible to conclude that, with the increase in concentration of POSS-Ph, the gas solubility improves. Additionally, the increase in coefficients *α^S^*_i/j_ indicates a more intensive increase in the solubility of CO_2_ than those of CH_4_ and N_2_. The improved CO_2_ sorption is connected with the presence of the phenyl groups present in the POSS-Ph structure [61]. Between CO_2_ molecules and aromatic hydrocarbons such as benzene, π–π interactions appear, which improve CO_2_ sorption. Additionally, in the case of this filler, sorption is strongly affected by the development of the specific surface of the membrane due to both the presence of filler particles and micro-cracks. On the other hand, on the basis of the results of the diffusion coefficients *D*_i_, a reduction in the diffusion rate of the gases through the membrane can be observed. This is probably due to an increase in the distance that the gas particles have to cover, owing to the presence of a nonporous filler. This effect limits the permeation of the test gases, despite the improvement of their solubility. On the other hand, due to the absence of a change in the degree of crystallinity of the membranes with 0.75 wt% and 2 wt% POSS-Ph content, it is possible to exclude adverse phenomena on the phase border in the material structure. The lack of results indicating the interactions between the filler particles and the polymer chains suggests that there are free phenyl substituents in the membrane. These groups, as in the case of their presence on a polymer surface, can interact with CO_2_ molecules, which in effect makes their transport difficult. The possible occurrence of this phenomenon is confirmed by the decrease in the values of the coefficients *α^D^*_i/j_, while the reduced crystallinity degree of the material for 8 wt% POSS-Ph concentration can be associated with the breaking of polymer chains due to the presence of filler particles. However, this effect is not as significant as to improve the diffusion of gases through the membrane. Changes in the membrane structure due to the presence of an inorganic filler can also be identified from TGA analysis. As with membranes with SiO_2_, the presence of POSS-Ph also increases the thermal strength of the sample. The increase in temperatures *T_d_*_5%_ and *T_d_*_10%_ confirms this effect. A slight decrease in the values of these parameters for a membrane with an additive content of 8 wt% may indicate that the bonds between the polymer chains break, which deteriorates the thermal strength of the structure. In conclusion, the decrease in the diffusion coefficient for membranes containing POSS-Ph is due to the extended transport distance that the gas molecules need to cover. On the other hand, the decrease in the coefficient *α^D^*_i/j_ may be associated with interactions between the phenyl groups and CO_2_ which slow down the diffusion of this gas.

## 4. Summary

To sum up the results and the discussion on them, it may be concluded that:

- There is a boundary concentration of a filler, above which the process properties of the membrane decrease. A further increase in the content of the dispersed phase also causes deterioration of the membrane’s mechanical properties;
- the presence of the test fillers improves the solubility of the test gases. This is connected with expanding the membrane surface, i.e., increasing the gas–membrane contact surface. This increase is even 2.13 times for membranes containing 8 wt% POSS-Ph in relation to pure polymer membranes. In addition, interactions occur between CO_2_ molecules and chemical groups connected with filler particles, which improve the sorption of this gas on the membrane surface. This effect improves the selectivity of the membrane. There was an increase in CO_2_/N_2_ selectivity of 121% for the membrane containing 2 wt% POSS-Ph;
- the presence of the dispersed phase reduces the selectivity of the membrane, taking into account only the ratios of diffusion coefficients, which is associated with the formation of defects in the material structure. The higher reduction (2.06 times) was noted for the membrane containing 10 wt% ZIF-8. For sufficiently high filler concentrations, these effects occur intensively enough to deteriorate the membrane selectivity. For initial concentrations of the additives, the adverse phenomena that emerge in the membrane structure—which may cause deterioration of the membrane selectivity—are reduced by a greater improvement in the solubility of CO_2_ than other gases;
- the tests confirmed the validity of selecting inorganic fillers, as they improve the process properties of the membranes;
- for the developed heterogeneous membranes, improvement of the process parameters (P, *α*) can be noticed. Considering the gas permeability through the membrane and the selectivity of the membrane, among the tested membranes, both for the separation of the CO_2_/CH_4_ mixture and the CO_2_/N_2_ mixture, the membrane made of Pebax^®^2533 and containing 5 wt% SiO_2_ seems to be the best.

## Figures and Tables

**Table 1:** DSC analysis results for membranes containing ZIF-8.

Parameter	PEBAX 2533	PEBAX 2533 + 2 wt% ZIF-8	PEBAX 2533 + 5 wt% ZIF-8	PEBAX 2533 + 10 wt% ZIF-8
*T_m_*_PE_ [°C]	16.4	16.1	14.6	15.0
*H_m_*_PE_ [J/g]	32.6	31.2	29.4	28.0
*X_C_*_PE_ [%]	16.3	15.6	14.7	14.0
*T_m_*_PA_ [°C]	141.0	140.7	137.9	138.3
*H_m_*_PA_ [J/g]	7.5	6.4	6.5	7.0
*X_C_*_PA_ [%]	3.3	2.8	2.8	3.0
***X_c_* [%]**	**13.7**	**13.0**	**12.3**	**11.8**

**Table 2:** DSC analysis results for membranes containing SiO_2_.

Parameter	PEBAX 2533	PEBAX 2533 + 2 wt% SiO_2_	PEBAX 2533 + 5 wt% SiO_2_	PEBAX 2533 + 10 wt% SiO_2_
*T_m_*_PE_ [°C]	16.4	16.0	15.7	15.2
*H_m_*_PE_ [J/g]	32.6	31.6	30.7	29.9
*X_C_*_PE_ [%]	16.3	15.8	15.3	14.9
*T_m_*_PA_ [°C]	141.0	140.8	140.5	140.2
*H_m_*_PA_ [J/g]	7.5	8.1	8.9	8.2
*X_C_*_PA_ [%]	3.3	3.5	3.9	3.6
***X_c_* [%]**	**13.7**	**13.3**	**13.0**	**12.7**

**Table 3:** DSC analysis results for membranes containing POSS-Ph.

Parameter	PEBAX 2533	PEBAX 2533 + 0.75 wt% POSS-Ph	PEBAX 2533 + 2 wt% POSS-Ph	PEBAX 2533 + 8 wt% POSS-Ph
*T_m_*_PE_ [°C]	16.4	16.6	15.4	15.9
*H_m_*_PE_ [J/g]	32.6	32.1	32.4	29.5
*X_C_*_PE_ [%]	16.3	16.0	16.2	14.7
*T_m_*_PA_ [°C]	141.0	140.0	140.3	139.7
*H_m_*_PA_ [J/g]	7.5	7.5	8.1	7.4
*X_C_*_PA_ [%]	3.3	3.3	3.5	3.2
***X_c_* [%]**	**13.7**	**13.5**	**13.7**	**12.4**

**Table 4:** Permeation coefficients *P*_i_ of the test gases for membranes of different ZIF-8 concentrations.

*C_m_*_ZIF-8_ [wt%]	0	2	5	10
*P*_CO2_ [barrer]	210.4 ± 1.7	272.5 ± 2.2	298.4 ± 2.4	437.2 ± 3.5
*P*_CH4_ [barrer]	37.2 ± 0.6	47.0 ± 0.8	55.0 ± 0.9	88.1 ± 1.4
*P*_N2_ [barrer]	11.7 ± 0.3	14.8 ± 0.4	18.0 ± 0.5	36.1 ± 0.9

**Table 5:** Coefficients of ideal selectivity *α*_i/j_ for membranes of different ZIF-8 concentrations.

*C_m_*_ZIF-8_ [wt%]	0	2	5	10
*α*_CO2/CH4_ [-]	5.66 ± 0.12	5.80 ± 0.12	5.43 ± 0.11	4.96 ± 0.10
*α*_CO2/N2_ [-]	17.98 ± 0.36	18.41 ± 0.37	16.58 ± 0.33	12.11 ± 0.24

**Table 6:** Permeation coefficients *P*_i_ of the test gases for membranes of different SiO_2_ concentrations.

*C_m_*_SiO2_ [wt%]	0	2	5	10
*P*_CO2_ [barrer]	210.4 ± 1.7	236.2 ± 1.9	258.6 ± 2.1	249.9 ± 2.0
*P*_CH4_ [barrer]	37.2 ± 0.6	38.0 ± 0.6	39.8 ± 0.6	40.7 ± 0.7
*P*_N2_ [barrer]	11.7 ± 0.3	12.2 ± 0.3	12.4 ± 0.3	12.8 ± 0.3

**Table 7:** Coefficients of ideal selectivity *α*_i/j_ for membranes of different SiO_2_ concentrations.

*C_m_*_SiO2_ [wt%]	0	2	5	10
*α*_CO2/CH4_ [-]	5.66 ± 0.12	6.27 ± 0.13	6.51 ± 0.13	6.14 ± 0.12
*α*_CO2/N2_ [-]	17.98 ± 0.36	19.45 ± 0.39	20.92 ± 0.42	19.52 ± 0.39

**Table 8:** Permeation coefficients *P*_i_ of the test gases for membranes of different POSS-Ph concentrations.

*C_m_*_POSS-Ph_ [wt%]	0	0.75	2	8
*P*_CO2_ [barrer]	210.4 ± 1.7	236.1 ± 1.9	240.2 ± 1.9	173.9 ± 1.4
*P*_CH4_ [barrer]	37.2 ± 0.6	40.2 ± 0.6	39.5 ± 0.6	36.3 ± 0.6
*P*_N2_ [barrer]	11.7 ± 0.3	11.6 ± 0.3	11.0 ± 0.3	10.7 ± 0.3

**Table 9:** Coefficients of ideal selectivity *α*_i/j_ for membranes of different POSS-Ph concentrations.

*C_m_*_POSS-Ph_ [wt%]	0	0.75	2	8
*α*_CO2/CH4_ [-]	5.66 ± 0.12	5.92 ± 0.11	6.08 ± 0.12	4.79 ± 0.10
*α*_CO2/N2_ [-]	17.98 ± 0.36	20.35 ± 0.41	21.82 ± 0.44	16.25 ± 0.33

## Appendix A

**Table A1:** Temperatures at which the weight of the sample decreases by 5 wt% and 10 wt% for a membrane containing different concentrations of ZIF-8.

	*T_d_*_5%_ [°C]	*T_d_*_10%_ [°C]
PEBAX 2533	345.1	369.5
PEBAX 2533 + 2 wt% ZIF-8	332.7	360.9
PEBAX 2533 + 5 wt% ZIF-8	324.1	357.0
PEBAX 2533 + 10 wt% ZIF-8	315.5	344.1

**Table A2:** Temperatures at which the weight of the sample decreases by 5 wt% and 10 wt% for a membrane containing different concentrations of SiO_2_.

	*T_d_*_5%_ [°C]	*T_d_*_10%_ [°C]
PEBAX 2533	345.1	369.5
PEBAX 2533 + 2 wt% SiO_2_	365.6	378.9
PEBAX 2533 + 5 wt% SiO_2_	364.3	379.4
PEBAX 2533 + 10 wt% SiO_2_	367.9	380.3

**Table A3:** Temperatures at which the weight of the sample decreases by 5 wt% and 10 wt% for a membrane containing different concentrations of POSS-Ph.

	*T_d_*_5%_ [°C]	*T_d_*_10%_ [°C]
PEBAX 2533	345.1	369.5
PEBAX 2533 + 0.75 wt% POSS-Ph	372.4	382.6
PEBAX 2533 + 2 wt% POSS-Ph	375.4	385.7
PEBAX 2533 + 8 wt% POSS-Ph	368.0	382.6

**Table A4:** Solubility coefficient *S*_i_ for membranes of different ZIF-8 concentrations. * The values in table should be multiplied.

*C_m_*_ZIF-8_ [wt%]	0	2	5	10
*S*_CO2_ *	0.375 ± 0.023	0.428 ± 0.025	0.465 ± 0.028	0.520 ± 0.031
*S*_CH4_ *	0.224 ± 0.008	0.216 ± 0.009	0.227 ± 0.010	0.235 ± 0.010
*S*_N2_ *	0.145 ± 0.009	0.145 ± 0.009	0.139 ± 0.009	0.158 ± 0.010

× [10^−3^ mol/(m^3^·Pa)].

**Table A5:** Diffusion coefficient *D*_i_ for membranes of different ZIF-8 concentrations. * The values in table should be multiplied.

*C_m_*_ZIF-8_ [wt%]	0	2	5	10
*D*_CO2_ *	1.88 ± 0.11	2.14 ± 0.12	2.22 ± 0.12	2.82 ± 0.16
*D*_CH4_ *	0.56 ± 0.01	0.76 ± 0.01	0.82 ± 0.01	1.10 ± 0.02
*D*_N2_ *	0.27 ± 0.02	0.34 ± 0.02	0.37 ± 0.03	0.77 ± 0.05

× [10^−6^ cm^2^/s].

**Table A6:** Coefficients *α*^S^_i/j_ and *α*^D^_i/j_ for membranes of different ZIF-8 concentrations.

*C_m_*_ZIF-8_ [wt%]	0	2	5	10
*α^S^*_CO2/CH4_ [-]	1.68 ± 0.08	1.98 ± 0.09	2.05 ± 0.09	2.21 ± 0.10
*α^S^*_CO2/N2_ [-]	2.58 ± 0.23	2.93 ± 0.26	3.33 ± 0.29	3.30 ± 0.40
*α^D^*_CO2/CH4_ [-]	3.38 ± 0.22	2.82 ±0.23	2.71 ± 0.21	2.56 ± 0.18
*α^D^*_CO2/N2_ [-]	6.96 ± 0.55	6.29 ± 0.50	6.00 ± 0.42	3.37 ± 0.27

**Table A7:** Solubility coefficient *S*_i_ for membranes of different SiO_2_ concentrations. * The values in table should be multiplied.

*C_m_*_SiO2_ [wt%]	0	2	5	10
*S*_CO2_ *	0.375 ± 0.023	0.521 ± 0.0312	0.536 ± 0.0322	0.598 ± 0.0200
*S*_CH4_ *	0.224 ± 0.008	0.310 ± 0.0096	0.325 ± 0.0101	0.319 ± 0.0064
*S*_N2_ *	0.145 ± 0.009	0.186 ± 0.0117	0.184 ± 0.0116	0.184 ± 0.0046

× [10^−3^ mol/(m^3^·Pa)].

**Table A8:** Diffusion coefficient *D*_i_ for membranes of different SiO_2_ concentrations. * The values in table should be multiplied.

*C_m_*_SiO2_ [wt%]	0	2	5	10
*D*_CO2_ *	1.88 ± 0.11	1.53 ± 0.09	1.62 ± 0.09	1.40 ± 0.08
*D*_CH4_ *	0.56 ± 0.01	0.41 ± 0.01	0.41 ± 0.01	0.43 ± 0.01
*D*_N2_ *	0.27 ± 0.02	0.22 ± 0.02	0.22 ± 0.02	0.23 ± 0.02

× [10^−6^ cm^2^/s].

**Table A9:** Coefficients *α*^S^_i/j_ and *α*^D^_i/j_ for membranes of different SiO_2_ concentrations.

*C_m_*_SiO2_ [wt%]	0	2	5	10
*α^S^*_CO2/CH4_ [-]	1.68 ± 0.08	1.68 ± 0.10	1.65 ± 0.10	1.88 ± 0.11
*α^S^*_CO2/N2_ [-]	2.58 ± 0.23	2.80 ± 0.25	2.91 ± 0.26	3.25 ± 0.29
*α^D^*_CO2/CH4_ [-]	3.38 ± 0.22	3.72 ± 0.22	3.94 ± 0.23	3.27 ± 0.19
*α^D^*_CO2/N2_ [-]	6.96 ± 0.55	6.95 ± 0.55	7.19 ± 0.57	5.81 ± 0.46

**Table A10:** Solubility coefficient *S*_i_ for membranes of different POSS-Ph concentrations. * The values in table should be multiplied.

*C_m_*_POSS-Ph_ [wt%]	0	0.75	2	8
*S*_CO2_ *	0.375 ± 0.023	0.482 ± 0.027	0.564 ± 0.034	0.799 ± 0.048
*S*_CH4_ *	0.224 ± 0.008	0.264 ± 0.007	0.270 ± 0.007	0.299 ± 0.009
*S*_N2_ *	0.145 ± 0.009	0.144 ± 0.008	0.148 ± 0.009	0.179 ± 0.011

× [10^−3^ mol/(m^3^·Pa)].

**Table A11:** Diffusion coefficient *D*_i_ for membranes of different POSS-Ph concentrations. * The values in table should be multiplied.

*C_m_*_POSS-Ph_ [wt%]	0	0.75	2	8
*D*_CO2_ *	1.88 ± 0.11	1.70 ± 0.10	1.45 ± 0.08	0.73 ± 0.04
*D*_CH4_ *	0.56 ± 0.01	0.51 ± 0.02	0.49 ± 0.02	0.40 ± 0.01
*D*_N2_ *	0.27 ± 0.02	0.27 ± 0.02	0.26 ± 0.02	0.20 ± 0.01

× [10^−6^ cm^2^/s].

**Table A12:** Coefficients *α*^S^_i/j_ and *α*^D^_i/j_ for membranes of different POSS-Ph concentrations.

*C_m_*_POSS-Ph_ [wt%]	0	0.75	2	8
*α^S^*_CO2/CH4_ [-]	1.68 ± 0.08	1.83 ± 0.14	2.09 ± 0.16	2.68 ± 0.17
*α^S^*_CO2/N2_ [-]	2.58 ± 0.23	3.35 ± 0.31	3.82 ± 0.37	4.46 ± 0.40
*α^D^*_CO2/CH4_ [-]	3.38 ± 0.22	3.33 ± 0.18	2.96 ± 0.14	1.79 ± 0.11
*α^D^*_CO2/N2_ [-]	6.96 ± 0.55	6.30 ± 0.45	5.80 ± 0.42	3.64 ± 0.29
